# Similarity solutions for early-time constant boundary flux imbibition in foams and soils

**DOI:** 10.1140/epje/s10189-021-00112-y

**Published:** 2021-08-31

**Authors:** Yaw Akyampon Boakye-Ansah, Paul Grassia

**Affiliations:** 1 Present Address: Department of Chemical and Process Engineering, University of Strathclyde, James Weir Building, 75 Montrose Street, Glasgow, G1 1XJ UK; 2 Department of Energy and Petroleum Engineering, University of Energy and Natural Resources, Sunyani, Ghana

## Abstract

**Abstract:**

The foam drainage equation and Richards equation are transport equations for foams and soils, respectively. Each reduces to a nonlinear diffusion equation in the early stage of infiltration during which time, flow is predominantly capillary driven, hence is effectively capillary imbibition. Indeed such equations arise quite generally during imbibition processes in porous media. New early-time solutions based on the van Genuchten relative diffusivity function for soils are found and compared with the same for drainage in foams. The moisture profiles which develop when delivering a known flux into these various porous materials are sought. Solutions are found using the principle of self-similarity. Singular profiles that terminate abruptly are obtained for soils, a contrast with solutions obtained for node-dominated foam drainage which are known from the literature (the governing equation being now linear is analogous to the linear equation for heat transfer). As time evolves, the moisture that develops at the top boundary when a known flux is delivered is greater in soils than in foams and is greater still in loamy soils than in sandstones. Similarities and differences between the various solutions for nonlinear and linear diffusion are highlighted.

**Graphic abstract:**

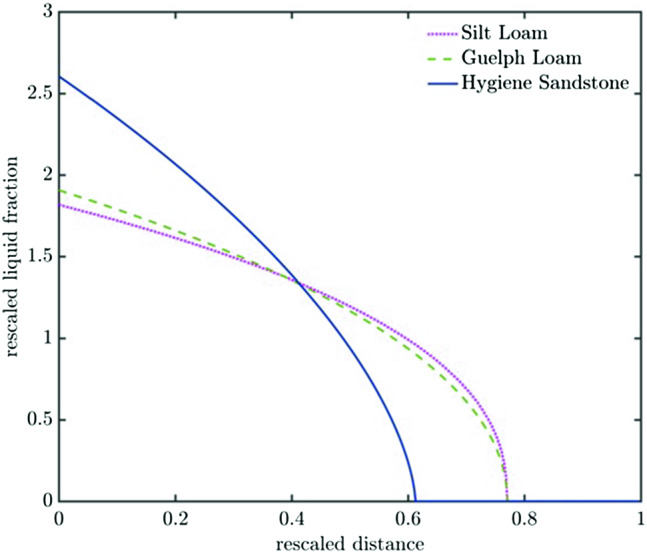

## Introduction

Infiltration in soils is an important problem that arises in multiple applications in agriculture, hydrology, soil sciences, and environmental engineering, among others [3, 4]. In work published previously [1], we presented solutions for the long-time propagation of moisture into porous media (soils or foams) which yields travelling waves as solutions of a convection–diffusion transport equation. In this present work, we focus instead on (nonlinear) diffusion equations as given for early-time or short-time infiltration (also called imbibition) processes. Early-time imbibition presents a rather different problem from general infiltration problems since the dominant driving force is a diffusive capillary force, instead of a balance between gravity and capillary diffusivity as happens subsequently, up to the point where the system is nearly fully saturated [1, 14]. Similarly for horizontal flow, the dominant force affecting flow is capillary diffusivity [14], and hence solutions obtained for early-time problems can describe this phenomenon as well. Meanwhile much later on, once full saturation is attained, the dominant force affecting flow of moisture in the soil is gravity. Thus, moisture evolution in porous media exhibits several different stages of behaviour during infiltration, but the early-time behaviour invariably involves (capillary) imbibition as we have said.

To summarise, at short times, diffusivity (due to capillary action) must dominate flow in soils since gravity-driven conduction of moisture is weak in dry systems. Indeed, gravity is negligible not only because the system is comparatively dry, but also because the gradients are realised over short distances at short time (strengthening the role of diffusivity). Hence, the governing equation for flow of moisture in soils, the so-called Richards equation [16], reduces to a porous medium nonlinear diffusion equation. Moreover, as alluded to earlier, in horizontal flow, the gravity term vanishes and reduces Richards equation to the same nonlinear diffusion equation [14, 21]. Thus, in either case we obtain a nonlinear diffusion problem. Self-similar forms of the moisture content $\varTheta$ in terms of position *x* and time *t* are then admitted. Various self-similar solutions have been obtained for this nonlinear problem for soils [8, 14, 21] and analogous systems [6]. All these various solutions recognise that the initial stage of moisture evolution into porous media describes entry from a source for very short times. The solutions therefore necessarily exhibit a large spatial gradient in moisture content over a small interval close to the source [22]. This structure is what is exploited to describe the infiltration process during early times, even though it gives way eventually to travelling waves at long times.

Soil material properties are required before any of the above-mentioned solutions can be obtained. Specifically, the properties that are required are the soil–water retention curve (SWRC), along with the relative hydraulic conductivity (RHC) and the relative diffusivity (RD) which derive from it. In the literature, a rather complicated soil-water retention curve (SWRC) proposed by van Genuchten [9] is commonly used, but since in the present work we will focus on the dry limit, it turns out to reduce to the simpler SWRC proposed by Brooks and Corey [5] (a power law). As a result, the SWRC, RHC and RD’ all reduce to power laws. Even with these simple material properties, the governing equation for evolution of moisture content remains nonlinear, and solutions of it (even self-similar ones) can usually only be obtained numerically.

Just as liquid can infiltrate into and flow through soils, it can also drain through foams [2, 10, 12, 20]. As far as the mathematical description of drainage is concerned, foams can be considered [7, 13] to have properties analogous to porous media such as soils. Mathematically speaking, the foam drainage equation can be regarded as a special case of Richards equation, just with very particular forms of the SWRC, RHC and RD [1]. Unlike other situations that we consider here, for foam drainage in the so-called node-dominated (ND) case [11, 12], relative diffusivity is unity at all liquid contents. This presents a special case for the early-time self-similar solution which can be solved analytically. Indeed, a linear diffusion equation results which is analogous to the linear equation for heat transfer. An alternative foam drainage case, the so-called channel-dominated (CD) case [19, 20] still however presents a diffusivity function which varies with moisture content, leading to a nonlinear diffusion equation.

The question that we address in this paper is, what is the evolving moisture content at the boundary of a system that is required to deliver a known imbibition flux into the system? Thus, we solve for a constant rate infiltration process. This changes the structure of the similarity solution relative to previous studies [8, 18, 22] which imposed a constant moisture content on a boundary. The scientific significance of the solution to be explored here is that when liquid flux is introduced to a soil it actually takes time for the moisture content at the top to grow [4] before eventually reaching a constant steady state [1]. The early-time solution explored here predicts how the moisture at the top would grow were it to be measured by a probe at the top say, and what the associated implications are for how deep moisture penetrates with time. Finding the solutions involves a two-point boundary value problem. We show that in foams, the moisture content on the boundary evolves more slowly with time than in soils. Moreover, as distance from the boundary increases, the moisture content of foam drainage variants go smoothly to zero whereas for the soils, this is abrupt, leading to a singularity at a location where the moisture content reaches zero. Thus, we manage to compare and contrast the behaviour of soils and foams, but focussing now on the early-time limit, unlike [1] which treated only long times.

The rest of this work is structured as follows. We consider the formulation of similarity solutions to the diffusion equation in the next section (Sect. 2). We consider two approaches to the solution, one using a “traditional” shooting method [15] and the other, still employing shooting, but using a flux as one of the solution variables. Novel features are introduced to the algorithm in order to deal with possible singularities. Section 3 meanwhile reviews the linear equations that derive from the diffusion equation with unit diffusivity, and Sect. 4 considers the nonlinear equations resulting from the presence of a variable (power law) diffusivity function. We discuss the solutions obtained in Sect. 5 and conclude the paper in Sect. 6.

## Formulation of early-time solutions

Richards equation [16] can be written in the form

1 $$\begin{aligned} (\theta _{s}-\theta _{r})\varTheta _t = -(K(\varTheta ))_{x} + (\theta _{s}-\theta _{r})(D(\varTheta )\varTheta _{x})_{x}. \end{aligned}$$

Here $\varTheta$ is a rescaled moisture content, which is defined as $\varTheta = (\theta -\theta _{r}) / (\theta _{s}-\theta _{r})$, with $\theta$ being moisture content (i.e. fraction of local pore space filled with liquid), $\theta _{r}$ being a residual moisture content, and $\theta _{s}$ being a saturation moisture content. Moreover, *x* is distance measured down from the top boundary, and *t* is time. Meanwhile, $K(\varTheta )$ is a hydraulic conductivity due to gravity, with $K(\varTheta )$ being a continuous function with the property that $K\rightarrow 0$ as $\varTheta \rightarrow 0$ and $K\rightarrow K_{s}$ (a saturation hydraulic conductivity) as $\varTheta \rightarrow 1$. In addition, $D(\varTheta )$ is a capillary diffusivity. This can be defined in terms of $K(\varTheta )$ and a capillary suction head $H(\varTheta )$ (also called a soil–water retention curve SWRC), such that $D(\varTheta )=K(\varTheta ) |\mathrm {d}H/\mathrm {d}\varTheta | \times (\theta _{s} - \theta _{r})^{-1}$.

The capillary suction head necessarily has some characteristic length scale (denoted $H_{c}$ say) associated with it. If we define relative hydraulic conductivity (RHC) as $K_{r}=K/K_{s}$ and relative diffusivity (RD) as $D_{r} = (\theta _{s}-\theta _{r})D/(K_{s}H_{c})$ and if we make lengths dimensionless on the scale $H_{c}$ and times dimensionless on the scale $(\theta _{s} - \theta _{r})H_{c}/K_{s}$, the above equation reduces to

2 $$\begin{aligned} \varTheta _t = -(K_{r}(\varTheta ))_{x} + (D_{r}(\varTheta )\varTheta _{x})_{x}, \end{aligned}$$

where for compactness of notation we now use *x* and *t* to denote dimensionless (rather than dimensional) distance and time, respectively.

In the early-time infiltration (or imbibition) limit, starting with a dry soil (or a dry foam), we expect values of $\varTheta$ to be small. Given that $K_{r}\rightarrow 0$ in the limit as $\varTheta \rightarrow 0$ [9], the following nonlinear diffusion equation then arises (it also arises in the case of horizontal flow without gravity)

3 $$\begin{aligned} \varTheta _t = \left( D_r \varTheta _x \right) _x. \end{aligned}$$

This is subject to the following conditions of the Dirichlet type at the end of the infiltration front, and of Neumann type at top surface boundary imposing a unit dimensionless flux at the top in all cases,

4 $$\begin{aligned}&\varTheta (x,t) \rightarrow 0, \ \text {as} \;\; x \rightarrow \infty , \qquad&D_r \varTheta _x = -1, \ \text {at} \;\; x = 0. \end{aligned}$$

Moreover, $$\varTheta (x,t)\rightarrow 0$$, as $$t\rightarrow 0$$.

As is evident from the form of Eq. (3), when moisture begins to accumulate in a porous medium, the infiltration in the early period is dominated by capillary suction. Hence, for the early-time solution, infiltration is independent of whatever the hydraulic conductivity might be, i.e. gravity-free suction into the porous medium occurs [4, 20]. As even more moisture accumulates over time however, the infiltration flux is partly due to capillary suction and partly due to hydraulic conductivity, the balance shifting from the former to the latter as time proceeds. We can still however use the early-time solution to estimate when the hydraulic conductivity might start to matter, a point we will return to later. In the first instance however, we focus on the capillary-dominated regime.

The diffusion equation can now be expressed as

5 $$\begin{aligned} \varTheta _{t} = (a\varTheta ^{N}\varTheta _{x} )_{x}, \end{aligned}$$

where $a\varTheta ^{N}$ represents the relative diffusivity function which in the dry limit is a power law function of $\varTheta$, raised to some power $N \ge 0$. Clearly for *N* strictly greater than zero, approaching the dry limit is challenging since diffusivity vanishes in that limit, and as we will see, the system responds by limiting the available domain for *x*.

It turns out that this power *N* has the value 0 and 1/2 for node-dominated and channel-dominated foam drainage, respectively [12, 20]. Meanwhile, for both node-dominated and channel-dominated foam drainage, the prefactor *a* is unity.

For soils, the values of *N* and *a* can be related to a soil-specific parameter *m* [9]. The value of *m* (with $0<m<1$) turns out to be a measure of what proportion of the volume tends to reside in very large pores, i.e. those that are much larger than the average for the specified medium. Values of *m* close to unity (as is typical for sandstones) have very little volume in pores much larger than average. Smaller values of *m* (typical for loams) mean it is less uncommon to find pores much larger than average, notwithstanding the fact that the average pore size in a loam tends to be smaller than in a sandstone. As we will see later on, *m* can also be related to the volume residing in pores that are much smaller than average. General formulae for $D_{r}(\varTheta )$ over a range of moisture contents have been given by [9] and taking these in the limit $\varTheta \ll 1$ gives $a = m-m^2$ and $N = 1/2+1/m$ (see [1] for details).

**Fig. 1:**
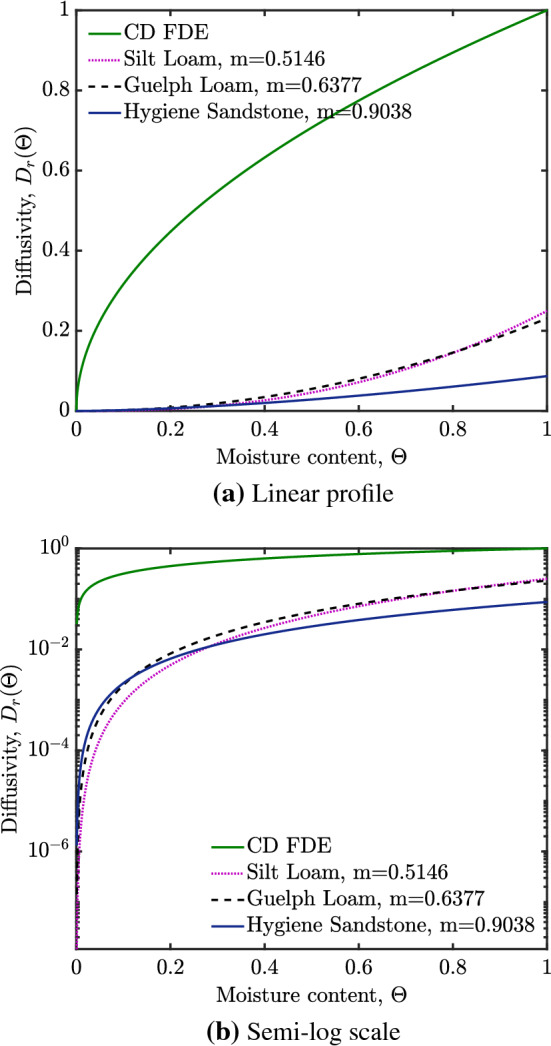
Diffusivity profile $D_r = a\varTheta ^N$ for the channel-dominated foam drainage equation (CD FDE) ($a=1$, $N=1/2$) and for three soil samples (with $a=m-m^2$, $N=1/2 + 1/m$, various *m*). The profile for node-dominated foam drainage equation (ND FDE) is not shown since it is unity at all moisture contents. The profile in (**a**) shows a linear plot while (**b**) is the same figure with $D_{r}$ on semilog scale to make it easier to see what happens at small $\varTheta$

Figure 1 shows the relative diffusivity profiles for channel-dominated foam drainage and three soil types. These are plotted here on the domain $0\le \varTheta \le 1$, although in the case of the soils what we have plotted are $\varTheta \ll 1$ limiting cases of formulae from [9] which are then extrapolated towards $\varTheta$ values beyond their limit of applicability. Such extrapolation is not problematic for the present work however since ultimately we will focus on small $\varTheta$ solutions. The $D_r$ profile for node-dominated foam drainage is not shown since it is unity for all $\varTheta$. As shown in the plot, at $\varTheta = 1$, relative diffusivity for (channel-dominated) foam drainage is also unity, but for the extrapolated [9] functions, the soils go to different $D_{r}$ values dependent on the prefactor which is $a = m-m^2$. We again observe that diffusivity at larger saturations is greater in loams than in sandstones, although the opposite is true when saturation is smaller: loams tend to have a higher value of $N=1/2+1/m$ and hence smaller $D_{r}$. For early-time infiltration, primarily we are interested in small $\varTheta$ values.

The origin of the factor $a= m - m^2$ can be traced back [1, 9] to the definition of $D_{r} = K_{r}|\mathrm {d}H_{+}/\mathrm {d}\varTheta |$ with $H_{+}\equiv H/H_{c}$ being a dimensionless suction head. The $K_r$ term contributes a factor $m^2$ to *a*, with the $|\mathrm {d}H_{+}/\mathrm {d}\varTheta |$ subsequently contributing the remaining factor $m^{-1}(1-m)$. The latter factor comes from $H_+$ being relatively insensitive to $\varTheta$ when *m* is close to unity, but much more sensitive when $m \ll 1$. The former factor (i.e. the $m^{2}$ factor) arises from systems with smaller *m* having a high proportion of their flux at full saturation coming from large pores. When we look at partial saturations in which only small pores tend to be filled, the flux in relative terms then becomes smaller.

The origin of the exponent $N=1/2+1/m$ meanwhile comes from summing two separate exponents [1, 9]. The first is an exponent $1/2+2/m$ appearing in $K_{r}$, representing again that at small *m*, comparatively little conduction occurs through the small pores. The second is an exponent $-1/m$ appearing in $|\mathrm {d}H_{+}/\mathrm {d}\varTheta |$ (representing that $H_{+}$ increases rapidly as $\varTheta$ decreases when *m* is small).

This completes the setting up of the early-time Richards equation, or analogously the early-time foam drainage equation. In the subsections to follow, we explain how to solve it in the particular constant rate infiltration (or imbibition) process that is considered here.

### Similarity equations

We consider that in the first instance the solution applies on a semi-infinite domain, since there is moisture infiltration or more specifically imbibition at one boundary whereas the moisture content $\varTheta$ approaches zero (dry limit) arbitrarily far from the boundary. In order to obtain solutions of Eq. (5) in a semi-infinite domain, we transform the independent variables in the equation into a self-similar form assuming the relationship

6 $$\begin{aligned} \varTheta (x,t)&= t^{1/(N+2)} \, \varPhi \left( \frac{x}{t^{(N+1)/(N+2)}} \right) ;&\eta {=} \frac{x}{t^{(N+1)/(N+2)}}, \end{aligned}$$

where the moisture content $\varTheta$ is represented by a similarity variable $\varPhi (\eta )$. Note the difference here from other studies [8, 14, 18, 22], which impose a fixed moisture content (as opposed to a fixed infiltration rate) on a boundary. Such solutions require a different similarity variable $x/t^{1/2}$ instead of $x/t^{(N+1)/(N+2)}$ as we have here. They also do not have the $t^{1/(N+2)}$ prefactor. However, in our case, it is this very prefactor which ensures that $\varTheta \ll 1$ in the early-time limit.

The variable $\eta$ chosen here (a function of both *x* and *t*) fulfils the similarity requirement. Imposing a unit flux at the boundary,

7 $$\begin{aligned} \left. a \varTheta ^{N}\varTheta _{x}\right| _{x= 0} = -1, \end{aligned}$$

then leads to

8 $$\begin{aligned} \left. a \varPhi ^{N}\varPhi '\right| _{\eta = 0} = -1. \end{aligned}$$

So far we have discussed just the boundary condition expressed in terms of the similarity coordinates. In order to solve the problem however, we need to transform the governing differential equation from a partial differential equation to an ordinary differential equation. This is achieved by expressing Eq. (5) in terms of the similarity expression deduced in Eq. (6), and leads to

9 $$\begin{aligned} (a \varPhi ^{N} \varPhi {'}){'} = \frac{\varPhi }{(N+2)} - \frac{(N+1)}{(N+2)} \eta \varPhi {'}. \end{aligned}$$

Equation (9) is a second-order equation, but typically we re-express it in terms of two first-order equations, one for the variation of $\varPhi$ and the other for the variation of $\varPhi '$. Even so, we require two boundary conditions. The boundary conditions in terms of the similarity variable are given as

10 $$\begin{aligned} \varPhi \rightarrow 0 \; \text {as} \; \eta \rightarrow \infty ; \qquad a\varPhi ^{N} \varPhi {'} = - 1 \; \text {as} \; \eta \rightarrow 0. \end{aligned}$$

This presents a two-point value boundary problem which we solve via the *shooting method*. Since Eq. (9) is a nonlinear equation, generally speaking a numerical method (such as shooting) is the only way of solving it [15]. Here, it is the initial value $\varPhi (0)$ that we seek via shooting, as the initial slope $\varPhi '(0)$ is then specified in terms of $\varPhi (0)$, as implied by the boundary conditions.

However, challenges still remain with solving the equations in their current form. Provided we have the correct $\varPhi (0)$ value, then as $\eta$ increases, we expect that both $\varPhi$ and the flux $-a\varPhi ^{N}\varPhi '$ will tend to zero. Depending on the parameter *N* however, it need not be the case that $\varPhi '$ approaches zero even though $\varPhi$ does. As we will see, it might be the case that $|\varPhi '|$ actually becomes infinite as $\varPhi \rightarrow 0$, but in such a way that the flux $-a\varPhi ^{N}\varPhi '$ still tends to zero. This then corresponds to moisture content falling to zero at a *finite* $\eta$ with an exceedingly abrupt approach of moisture content towards zero. This is a behaviour that is also seen in long-time travelling wave solutions of Richards equation [1]. Although those long-time travelling wave solutions are by no means the same as the diffusive, early-time similarity solutions considered here, analogies still apply. Sufficiently close to the front of a travelling wave, any flux that is present can be shown to be dominated by a capillary diffusivity (even though, further back the flux in the travelling wave is gravity dominated).

Returning now to consider the early-time case which is to be tackled via a shooting method, if we choose a too large value of $\varPhi (0)$, the value of $\varPhi$ typically fails to fall to zero at all. Instead $\varPhi$ reaches a minimum and starts to increase again, at large $\eta$ becoming proportional to $\eta ^{1/(N+1)}$. It is easy to check that this functional form $\varPhi \sim \eta ^{1/(N+1)}$ is a solution of Eq. (9), albeit not the solution we seek. On the other hand, if we choose a too small value of $\varPhi (0)$, then $\varPhi$ typically falls to zero abruptly, but now with $|\varPhi '|$ becoming infinite very rapidly, such that $-a\varPhi ^{N}\varPhi '$ remains finite: again this is not the solution we seek. As we will see however for the correctly chosen $\varPhi (0)$, whereas $\varPhi$ still falls to zero abruptly and whereas $|\varPhi '|$ still can become infinite, the divergence of $\varPhi '$ is sufficiently slow that $-a\varPhi ^{N}\varPhi '$ still manages to approach zero.

We are faced then with the challenge of needing to distinguish between two solutions for which $|\varPhi '|$ diverges, i.e. a rapid divergence (an incorrectly chosen $\varPhi (0)$) and a slower divergence ($\varPhi (0)$ chosen correctly). Dealing with these divergent quantities in a numerical scheme is not straightforward. A simpler approach however is to replace the unknown $\varPhi '$ by a flux variable which turns out to better behaved in the limit as $\varPhi \rightarrow 0$. This approach is explained next.

### Flux-based solution

The flux, *F* (represented in terms of similarity variables), can be expressed as

11 $$\begin{aligned} {F} = -a \varPhi ^N \varPhi '. \end{aligned}$$

Here flux is positive in general since $\varPhi '<0$ in solutions of interest.

Equation (9) can therefore be rewritten in terms of *F* as

12 $$\begin{aligned} {F'} = -\frac{\varPhi }{N+2} - \frac{(N+1)}{(N+2)}\frac{\eta F}{a\varPhi ^N}. \end{aligned}$$

These two equations (11) (rearranged into the form $\varPhi ' = - F / (a \varPhi ^{N})$) & (12) now provide two first order differential equations in two unknowns $\varPhi$ and *F*.

The boundary conditions for this problem are given in Eq. (10), although the second boundary condition can now be expressed more elegantly as $F(0)=1$. We now seek, via the shooting method, a $\varPhi (0)$ value such that $\varPhi$ and *F* vanish simultaneously, either at $\eta \rightarrow \infty$ or else for some finite but as yet unspecified maximum $\eta$ value, denoted $\eta _{\max }$.

In the case where $\varPhi$ and *F* vanish simultaneously at some finite $\eta =\eta _{\max }$, they will both remain identically zero for all $\eta >\eta _{\max }$ [18]. This means in particular that $\varTheta (x,t)$ will be identically zero for any *x* greater than some value $x_{\max }$, where we define $x_{\max } \equiv \eta _{\max }\,t^{(N+1)/(N+2)}$.

The code implementing the shooting solution was written in-house using MATLAB. We employed the fourth-order Runge–Kutta method to solve the system of equations, using a step-size for $\eta$ of $\texttt {0.001}$, and thereby determined whether a selected $\varPhi (0)$ needed to be increased or decreased to satisfy boundary conditions, the correct $\varPhi (0)$ being found via a bisection approach. The obtained Runge–Kutta solutions were compared with the inbuilt routine ode45. The solutions ultimately obtained for $\varPhi (0)$ agreed within four significant figures or better, regardless of whether we considered variables $\varPhi$ and $\varPhi '$ or variables $\varPhi$ and *F*.

In order to benchmark our numerical scheme, we carried out additional tests. Firstly, we solved the node-dominated equation (see Eq. (21) given in Sect. 3.1) via the numerical scheme but using the value of $\varPhi (0)$ obtained from an analytical solution (Eq. (21), being linear, admits an analytical solution Eq. (22)). Having obtained a satisfactory solution for $\varPhi$ versus $\eta$ via this benchmark, we then applied the numerical scheme to nonlinear systems, obtaining the results shown in Sect. 4. Before any of that though, we describe in the subsections to follow, how the algorithm we used overcame some of the challenges presented by possibly singular solutions.

### Behaviour of flux when $\varvec{\varPhi \rightarrow }$ 0 at a finite $\varvec{\eta =\eta _{\max } }$

The equations written in flux form enable us to distinguish between the cases in which $\varPhi (0)$ is selected too small within the shooting algorithm, meaning that $\varPhi$ approaches zero at some finite $\eta _{\max }$ still with a finite flux *F* there, and the case in which $\varPhi (0)$ is selected correctly, meaning both $\varPhi$ and *F* vanish at $\eta _{\max }$. Cases in which $\varPhi (0)$ is selected too large do not need special consideration, since $\varPhi$ never reaches zero anywhere in the solution domain in that case, so bespoke techniques to analyse the limiting behaviour as $\varPhi \rightarrow 0$ are not needed.

Close to $\varPhi \rightarrow 0$, it is clear that the second term on the right-hand side Eq. (12) dominates the first term on the right. Hence, $F'$ is approximated close to $\eta _{\max }$ by

13 $$\begin{aligned} F' \approx - \frac{(N+1)}{(N+2)} \frac{F \eta _{\max }}{a \varPhi ^{N}} = \frac{(N+1)}{(N+2)} \varPhi ' \eta _{\max }, \end{aligned}$$

from which it follows (using $F_{m}$ to denote the value of *F* at $\eta =\eta _{\max }$),

14 $$\begin{aligned} F - F_{m} \approx \frac{(N+1)}{(N+2)} \varPhi \eta _{\max }. \end{aligned}$$

The generic case we consider is that close to $\eta _{\max }$,

15 $$\begin{aligned} \varPhi&\sim \varepsilon (\eta _{\max } - \eta )^{1/(N+1)},\nonumber \\ \varPhi '&\sim - \varepsilon (\eta _{\max } - \eta )^{-N/(N+1)}/(N+1), \end{aligned}$$

where $\varepsilon$ is some a priori unknown value. The value of $F=-a\varPhi ^{N}\varPhi '$ close to $\eta =\eta _{\max }$ becomes

16 $$\begin{aligned} F \rightarrow F_{m} = \frac{a \varepsilon ^{N+1}}{(N+1)}, \end{aligned}$$

which indicates that power law relations in (15) are exactly the ones required to admit finite flux even though $\varPhi \rightarrow 0$.

In practice, we use the above equations as follows. Suppose using a numerical Runge–Kutta scheme, we know values of both $\varPhi$ and *F* at some value of $\eta$, and also that $\varPhi \ll 1$ at this point. Then we can use Eq. (15) to eliminate $\varepsilon$ in favour of $\varPhi$ and $\eta _{\max }-\eta$. We substitute that expression in (16) to determine a formula for $F_{m}$,

17 $$\begin{aligned} F_{m} \approx \frac{a}{(N+1)} \frac{\varPhi ^{N+1}}{(\eta _{\max }-\eta )}, \end{aligned}$$

and finally substitute this into (14). This then leads to an expression for $\eta _{\max }$ given the values of $\varPhi$ and *F* at some nearby $\eta$. In other words, we have estimated the point at which $\varPhi$ is exactly zero, starting from a point at which $\varPhi$ is nearly zero. The value of $F_{m}$ then follows, but generally is nonzero, indicating an incorrectly chosen $\varPhi (0)$, meaning a different $\varPhi (0)$ value must be sought. How this scheme changes when $\varPhi (0)$ is eventually chosen correctly is what we discuss next.

### Behaviour when $\varPhi \rightarrow 0$ & $F\rightarrow 0$ at $\eta =\eta _{\max }$

For a correctly chosen $\varPhi (0)$, we anticipate that $F_{m}$ should approach zero. In that case, we anticipate a power law behaviour,

18 $$\begin{aligned} \varPhi \sim \varepsilon (\eta _{\max }-\eta )^{1/N},\ \ \varPhi ' \sim - \varepsilon (\eta _{\max }-\eta )^{(-1 + 1/N)}/N. \end{aligned}$$

Note that if $N>1$, this corresponds to an abrupt approach of $\varPhi$ to zero, in the sense that $|\varPhi '|\rightarrow \infty$. Even so, $|\varPhi '|$ does not diverge as rapidly as what we saw previously in Eq. (15).

Assuming Eq. (18) applies, it then follows via Eq. (11) that,

19 $$\begin{aligned} F \approx a \varepsilon ^{N+1} (\eta _{\max } - \eta )^{1/N} / N. \end{aligned}$$

Note that the ratio $F/\varPhi$ is therefore $a \varepsilon ^{N} / N$, i.e. it is insensitive to exactly how close $\eta$ is to $\eta _{\max }$. However, Eq. (14) with $F_{m}\rightarrow 0$ suggests this ratio $F/\varPhi$ is nothing more than $(N+1)\eta _{\max }/(N+2)$. If we equate these quantities and now use Eq. (18) to eliminate $\varepsilon$ in favour of $\varPhi$ and $\eta _{\max }-\eta$ we obtain

20 $$\begin{aligned} \frac{a \varPhi ^{N}}{(\eta _{\max }-\eta )N} \approx \frac{(N+1)\eta _{\max }}{(N+2)}. \end{aligned}$$

We use this equation as follows. Suppose for a certain $\eta$, the values of $\varPhi$ and *F* are finite, but both are small, such that $\varPhi \ll 1$ and $F\ll 1$. Equation (20) is solved for $\eta _{\max }$. We then verify a posteriori that the flux evaluated at $\eta _{\max }$, which is estimated via Eq. (14) as $F-(N+1)\varPhi \eta _{\max }/(N+2)$, is much smaller than *F* itself.

A number of observations follow. Recall *N* is the exponent of the power law relating capillary diffusivity to moisture content, in the case of soils taking the value $N = 1/2 + 1/m$ for a soil specific parameter $0<m<1$. For any $N>1$, Eq. (18) indicates an abrupt approach of moisture content towards zero. Specifically moisture content scales like distance from the leading edge of the front raised to the power 1/*N*. Equation (18) was derived here in the context of early-time similarity solutions, but (as already alluded to previously) the exact same power law relating moisture content and distance was found [1] for long-time travelling waves.

Returning to the early-time case, the situation we solve for channel-dominated foam drainage is different from soils, since the former has $N={1}/{2}$. Equation (18) still predicts a $\varPhi$ value that tends to zero at a finite $\eta _{\max }$ but the approach is not abrupt, since Eq. (18) now gives moisture content varying like the square of distance from the leading edge (again the same behaviour is observed close to zero moisture content in the long-time channel-dominated case [1]).

The node-dominated foam drainage case has $N=0$, and Eq. (18) then ceases to apply. In fact, in the node-dominated case there is no finite $\eta _{\max }$ at which moisture content vanishes. Instead we only ever have $\varPhi \rightarrow 0$ when $\eta \rightarrow \infty$. As a matter of fact, the $N=0$ case is simple to solve since it has constant diffusivity and so the governing differential equation now becomes linear. This situation is discussed shortly (see Sect. 3).

It is also possible in principle to consider a foam drainage situation which is intermediate between the node-dominated and channel-dominated cases, with $0<N<1/2$. This should admit solutions to Eqs. (18)–(19) with a finite $\eta _{\max }$. As also happens in the channel-dominated case (with $N=1/2$), this intermediate situation with $0<N<1/2$, taking the limit $\eta \rightarrow \eta _{\max }$, should allow $\varPhi$ to decay to zero gradually, rather than abruptly. The governing differential equation for $\varPhi$ still however remains nonlinear if this intermediate situation is considered.

## Linear equation: constant diffusivity

As earlier noted, based on the diffusivity function for node-dominated foam drainage, a linear differential equation for moisture content is obtained. We discuss this situation below.

### Node-dominated foam drainage

In the node-dominated case ($N=0$ and $a=1$), Eq. (5) reduces to $\varTheta _t = \varTheta _{xx}$. Equation (6) now suggests looking for a solution of the form $\varTheta = t^{1/2}\varPhi (\eta )$ with $\eta =x/t^{1/2}$. Here $\varPhi$ must satisfy (as follows from Eq. (9) if we set $N=0$)

21 $$\begin{aligned} 2\varPhi {''} + \eta \varPhi {'} - \varPhi = 0. \end{aligned}$$

The solution of this turns out to be [6]

22 $$\begin{aligned} \varPhi (\eta ) = \ -\eta \,{\mathrm{erfc}} \left( {}^{\eta }\!/_{2} \right) + \frac{2}{\sqrt{\pi }} \exp {(-{}^{\eta ^2}\!/_{4})}. \end{aligned}$$

**Fig. 2:**
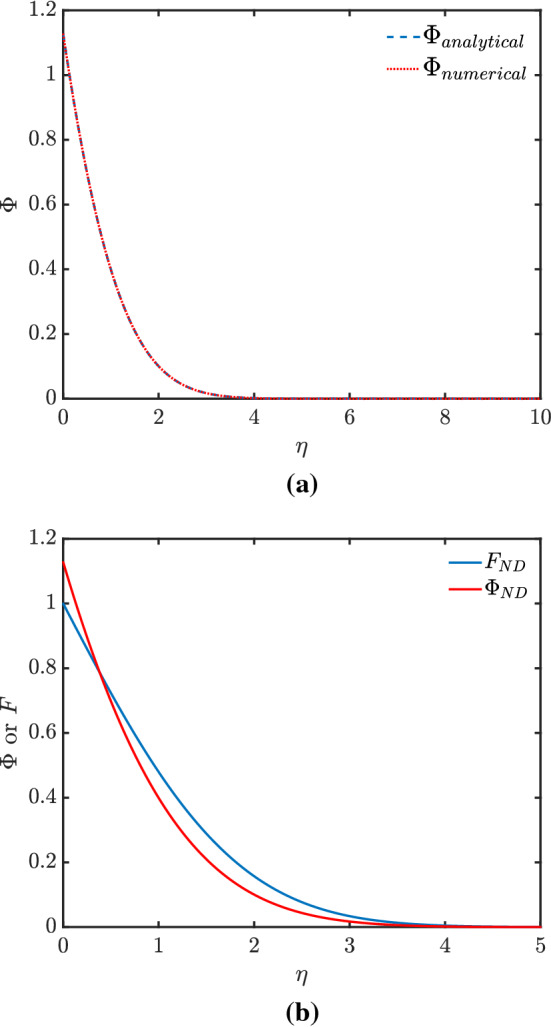
Profiles for node-dominated (ND) foam drainage showing (**a**) comparison of $\varPhi$ computed by numerical method and analytical solution, and (**b**) values of $\varPhi$ and *F*

The flux *F* is now simply $-\varPhi '$ with $\varPhi ' = -\,{\mathrm{erfc}} (\eta /2)$, so it is easy to check that the correct boundary condition $F=1$ at $\eta \rightarrow 0$ is obtained. It follows that the correct value of $\varPhi (0)$ we seek is $2/\sqrt{\pi }\approx 1.128$. Knowing this $\varPhi (0)$ value in advance in this case $N=0$ allows us to benchmark the numerical shooting method mentioned in Sect. 2.2. In Fig. 2a, we compare the analytical solution for $\varPhi$ with the numerical one to verify they match. Meanwhile, in Fig. 2b we plot $\varPhi$ and *F* computed from the analytical solution.

Note in Fig. 2b at large $\eta$, that $\varPhi$ itself decays much more quickly than *F* does. For large $\eta$, there is an approximate asymptotic formula for $\,{\mathrm{erfc}}$ [6], from which it follows,

23 $$\begin{aligned} F= \,{\mathrm{erfc}} \left( \frac{\eta }{2} \right)&\sim \exp \left( \frac{-\eta ^2}{4} \right) \frac{2}{\eta \sqrt{\pi }} \left( 1 - \frac{2}{\eta ^2}\right) , \end{aligned}$$

which via Eq. (22) leads in turn to, still in the large $\eta$ limit

24 $$\begin{aligned} \varPhi&\sim \exp \left( -\frac{\eta ^2}{ 4}\right) \times \frac{4}{\sqrt{\pi } \eta ^2}, \end{aligned}$$

which is order $2/\eta$ times smaller than *F*. It follows from this that $\varPhi$ decays much more quickly at large $\eta$ than either term $\eta \,{\mathrm{erfc}} (\eta /2)$ or $({2}/{\sqrt{\pi }})\exp (-{}^{\eta ^2}\!/_{4})$ that contributes to $\varPhi$ decays, i.e. at large $\eta$, the two terms contributing to $\varPhi$ almost (but not quite) cancel one another out.

This completes our analysis of the linear equation that arises when $$ N = 0, $$ this analysis being known from literature [6]. In the following section, we present results from nonlinear equations with nonzero *N*, the results to be presented now being obtained via the methods already outlined in Sects. 2.1–2.4.

## Nonlinear equations: variable diffusivity

As stated earlier, by the nature of their diffusivity functions, the diffusion equation for the channel-dominated foam drainage and Richards equations are nonlinear. The diffusivity functions turn out to be $\varTheta ^{1/2}$ and $(m-m^2)\varTheta ^{1/2+1/m}$, respectively, for channel-dominated [19] foam drainage and Richards [16] equation, in the latter case diffusivities having been obtained via the expressions from [9]. As already mentioned, we write these diffusivities generically as $D_{r} = a \varTheta ^{N}$.

### Channel-dominated foam drainage

**Fig. 3:**
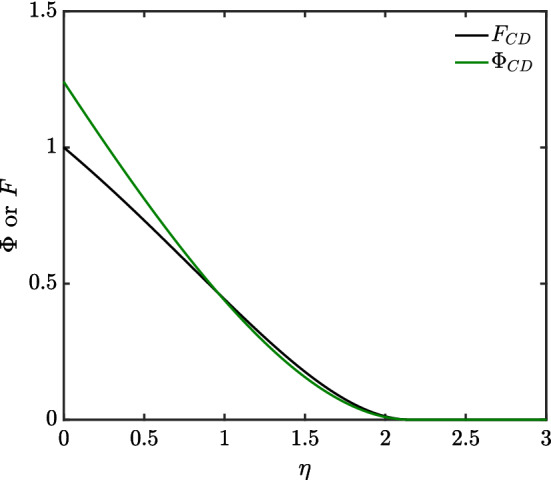
Profile of channel-dominated (CD) foam drainage $\varPhi$ and *F* via shooting method

Data obtained from the shooting method in the channel-dominated case ($N=\frac{1}{2}$) are presented in Fig. 3. We consider data both for $\varPhi$ and *F*.

Notice that (as discussed in Sect. 2.4) $\varPhi$ and *F* both go to zero at a finite $\eta _{\max }$, but the approach is gradual rather than abrupt. The value of $\varPhi '$ also tends to zero as $\eta \rightarrow \eta _{\max }$ but the approach (albeit not plotted here) is less gradual than that of either $\varPhi$ or *F*.

**Table 1:** Values of $\varPhi (0)$ and $\eta _{\max }$ for various porous media (namely node-dominated foam, channel-dominated foam and three types of soil). Values of $\varPhi (0)$ subscripted ‘*SM*’ are from the original “traditional” shooting method in terms of $\varPhi$ and $\varPhi '$ (rather than in terms of $\varPhi$ and *F* which is the preferred method here). The value for the ND foam is an exact analytical solution

Porous medium	*m*	*a*	*N*	$\varPhi (0)$	$\varPhi (0)_{SM}$	$\eta _{\max }$
Node-dominated foam	–	1	0	$2/\sqrt{\pi }$	–	–
Channel-dominated foam	–	1	1/2	1.2410	1.2410	2.1587
Silt Loam	0.5146	0.2498	2.4433	1.8183	1.8186	0.7695
Guelph Loam	0.6377	0.2310	2.0681	1.9074	1.9077	0.7698
Hygiene Sandstone	0.9038	0.0869	1.6064	2.6065	2.6066	0.6119

Table 1 gives the estimated $\varPhi (0)$ that satisfies the boundary conditions above. This value is greater than the node-dominated $\varPhi (0)$ value. This is as expected because it is possible to show that $\int _{0}^{\eta _{\max }^{*}} \varPhi \,\mathrm {d}\eta =1$ where $\eta _{\max }^{*}$ either equals $\eta _{\max }$ (if there is a finite $\eta _{\max }$ value at which $\varPhi =F=0$) or else is infinity (if $\varPhi$ and *F* only decay to zero as $\eta \rightarrow \infty$). Hence, a smaller value of $\eta _{\max }^{*}$ tends to be compensated by a larger value of $\varPhi (0)$ to keep the value of the integral fixed. This integral relation follows from the notion that if a unit flux enters the system at the top boundary and is accumulated over a time *t*, then $\int _{0}^\infty \varTheta \,\mathrm {d}x = t$, as indeed follows from integrating Eq. (5) over *x* and *t*. The same relation also follows by writing Eq. (9) in the form,

25 $$\begin{aligned} (a \varPhi ^{N}\varPhi ')' = \varPhi - \frac{(N+1)}{(N+2)} (\eta \varPhi )', \end{aligned}$$

and then integrating from 0 to $\eta _{\max }^{*}$.

The data obtained from the solution are given in Table 1. The method formulating the equations in terms of $\varPhi$ and *F* (Sect. 2.2) has been checked against the method formulating them in terms of $\varPhi$ and $\varPhi '$ (Sect. 2.1), and both methods give the same data to at least 4 significant figures. Here $\varPhi (0)\approx 1.2410$ for the channel-dominated foam drainage equation, and this exceeds the corresponding node-dominated value of $2/\sqrt{\pi }\approx 1.128$.

### Richards equation: van Genuchten diffusivity function

We focus on the solution of the early-time diffusion problem for soils using the relative diffusivity functions of van Genuchten [9] but taken in the small $\varTheta$ limit recognising here that at early-times one only ever accesses small $\varTheta$. As was the case in Sect. 4.1, there is no general closed-form solution for this problem, so we solve it numerically as a two-parameter shooting problem using the approach discussed in Sect. 2.2. In what follows, we show the numerical solution for $\varPhi (\eta )$. Note that $\varPhi$ now approaches zero (abruptly) as $\eta \rightarrow \eta _{\max }$ (for a finite $\eta _{\max }$), and it stays zero for all $\eta >\eta _{\max }$.

The solutions vary for different soil types according to the value of *m*, since each soil type has its own form of $D_{r}(\varTheta )$ which dictates early-time spreading. These different $D_{r}$ functions affect how much moisture accumulates near the top of the soil and how far into the soil it propagates at early times. Each soil type thereby has a different $\varPhi (0)$ and different $\eta _{\max }$. The value of $\varTheta (0,t)$ and the maximum penetration distance in *x* (denoted $x_{\max }$ say) then obey $\varPhi (0)t^{1/(N+2)}$ and $\eta _{\max }t^{(N+1)/(N+2)}$, where recall $N=1/2 + 1/m$.

**Fig. 4:**
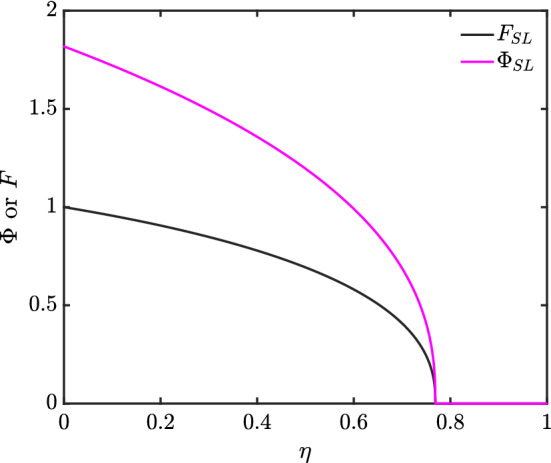
Profiles of $\varPhi$ and *F* for Silt Loam (SL), $m = 0.5146$

**Fig. 5:**
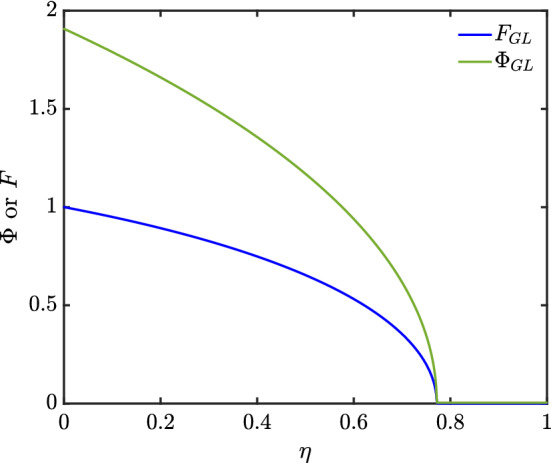
Profiles of $\varPhi$ and *F* for Guelph Loam (GL), $m = 0.6377$

**Fig. 6:**
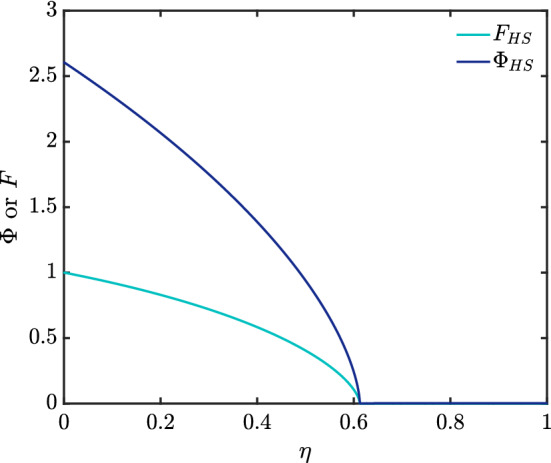
Profiles of $\varPhi$ and *F* for Hygiene Sandstone (HS), $m = 0.9038$

**Fig. 7:**
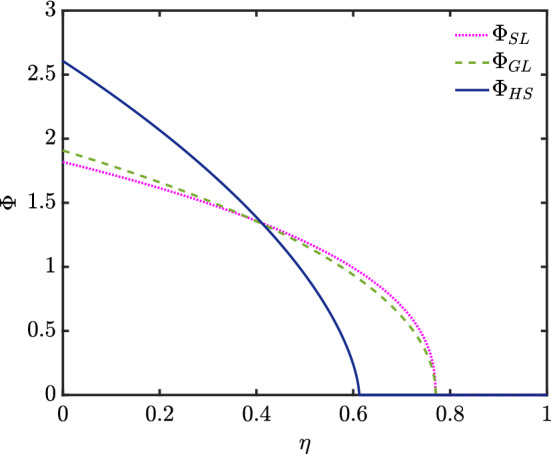
Profile showing $\varPhi$ versus $\eta$ for the three soil types: Silt Loam (SL), Guelph Loam (SL) and Hygiene Sandstone (HS)

The solutions for $\varPhi (\eta )$ and $F(\eta )$ are shown in Fig. 4–6 for three different soil types, and the $\varPhi (\eta )$ profiles are combined together for easy comparison in Fig. 7. Considering Eq. (9) in the case of Hygiene Sandstone (HS) ($m = 0.9038$) and using the boundary conditions given in Eq. (10), we obtain $\varPhi (0) = 2.6065$ as shown in Table 1. The data for the other soils (Silt Loam (SL) $m=0.5146$ and Guelph Loam (GL) $m=0.6377$) have smaller $\varPhi (0)$, namely 1.8183 and 1.9074, respectively. What is striking in Table 1 is that the two loams have nearly the same $\eta _{\max }$ despite having different $\varPhi (0)$. The areas under the $\varPhi$ versus $\eta$ curves are all the same however regardless of how $\varPhi (0)$ and $\eta _{\max }$ vary.

## Discussion

We have thus far solved for various nonlinear cases including channel-dominated foam drainage and flow in soils, the solutions of which are shown in Fig. 3–6, and Table 1. We expect these solutions are unique, since [17, 18] showed that nonlinear diffusion problems with singularities typically have only one solution. Additionally, in [18], it was postulated that if $D_{r}(\varTheta )> 0$ for $\varTheta >0$ but $D_{r}(0)=0$, singular behaviour consisting of $\varTheta (\eta )$ vanishing at some finite point $\eta _{\max }$ and then $\varTheta (\eta )$ vanishing identically $\eta \ge \eta _{\max }$ is possible: this is indeed the behaviour that we see. Although the similarity expression used in that publication is not what we employ due to the different problem we solve (i.e. a constant boundary flux rather than a constant boundary moisture content), the principle still follows.

In what follows, we use our results to look specifically at the temporal evolution of the system.

### Estimating moisture content at the top boundary at early times

We know that $\varTheta (x,t) = t^{1/(N+2)}\varPhi (\eta )$ and hence $\varTheta (0,t)=\varPhi (0)t^{1/(N+2)}$. When we substitute in for the soil specific parameter *m* in lieu of $N=1/2 + 1/m$, we find $\varTheta (0,t) = \varPhi (0)t^{2m/(2 + 5 m)}$. The power law exponent tends to indicate that at early times ($t\ll 1$), $\varTheta (0,t)$ for loams (smaller *m*) grows faster than for sandstones (or indeed for foams which have an even higher exponent still). Offsetting this is the fact that the prefactor $\varPhi (0)$ tends to be smaller in loams than in sandstones.

This tells us how moisture content grows at the top boundary, assuming constant unit flux there, and assuming also the flux is entirely diffusive. In reality though, $F = K_r (\varTheta ) - D_r(\varTheta ) \varTheta _x$, i.e. any imposed flux will be comprised of both a conductive and diffusive part. If the value of $\varTheta$ at the boundary is estimated as $\varPhi (0)t^{2m/(2+5m)}$, it is possible to estimate how the conductive flux which is $K_{r}\approx m^{2}\varTheta ^{1/2 + 2/m}$ in the small $\varTheta$ limit [1] evolves at the top boundary. If the estimated $K_{r}(\varTheta )$ becomes too large, then the assumption we have made that flux is dominated by diffusion ceases to be valid. In the early-time limit though, we end up with $K_{r}(0,t) \approx m^{2}(\varPhi (0))^{1/2+2/m} t^{(4+m)/(2 + 5 m)}$. The power law now indicates that for $t \ll 1$, $K_{r}$ grows more slowly for loams (small *m*) than for sandstones (larger *m*), even though $\varTheta (0,t)$ is faster growing in the case of loams. This is compounded by the factor $m^2 (\varPhi (0))^{{1/2+2/m}}$ which is smaller in loams.

We now estimates three particular times $t_{\varTheta (0,t)\sim 0.1}$, $t_{K_{r}(0,t)\sim 0.1}$, $t_{\varTheta (0,t)\sim 1}$, for soils and for foam drainage, and present these in Table 2. The first quantity is the time at which moisture content at the top boundary starts to become significant (reaching an estimated value $\varTheta \approx 0.1$, i.e. just an order of magnitude less than full saturation). The second is the estimated time at which conductive flux at the top (which is neglected from our purely diffusive model) would start to become significant, i.e. $K_{r}\approx 0.1$ compared with the total flux $F=1$. The $\varTheta$ value at the top attained at this time we denote $\varTheta _{K_{r}(0,t)\sim 0.1}$. The third time that we identify is the time at which moisture content at the top boundary would be predicted via the purely diffusive model to be so large $\varTheta \approx 1$, that the model has certainly broken down. Indeed, if we ever obtain $\varTheta \approx 1$ at the top boundary then since the relative hydraulic conductivity $K_{r}$ approaches unity (by construction) in the $\varTheta \rightarrow 1$ limit, the flux will definitely be dominated by conduction not diffusion (this is the long-time limit identified by [1]). Note that the soil material property functions we have employed $K_{r}\approx m^{2} \, \varTheta ^{1/2+2/m}$ and $D_{r}\approx m^{-1}(1-m)\varTheta ^{1/2+1/m}$ are unreliable in this limit (we used $\varTheta \ll 1$ approximations to the material functions of [9] here). In particular, if we extrapolate the $\varTheta \ll 1$ formula, $K_{r}\approx m^{2} \, \varTheta ^{1/2+2/m}$ all the way to $\varTheta \rightarrow 1$, we do not obtain $K_{r}\rightarrow 1$. Nevertheless, the original material functions [9] do actually give $K_{r}\rightarrow 1$ in this limit. In any case, as already mentioned, the early-time similarity solution for $\varTheta$ versus *x* and *t* is replaced by a long-time travelling wave in this case.

Table 2 reveals that loams attain $\varTheta (0,t)\approx 0.1$ very quickly indeed, followed by sandstones, with foams requiring more time still. The reason loams attain $\varTheta (0,t)\approx 0.1$ so quickly follows from the relation for $\varTheta (0,t)$ that was given earlier $\varTheta (0,t) \approx \varPhi (0) t^{2m/(2+5m)}$. Upon rearrangement, this gives $t\approx (\varTheta (0,t)/\varPhi (0))^{5/2 +1/m}$. Notwithstanding the fact that different soil types with different *m* values have different $\varPhi (0)$, the main effect here when we set $\varTheta (0,t)\approx 0.1$ is that *t* is obtained by raising a small number to a high power when *m* is small (as happens for loams). The estimated time to approach $\varTheta (0,t)\approx 1$ (as opposed to $\varTheta (0,t)\approx 0.1$) is also much less in soils than in foams, but sandstones now attain this quicker than loams (which is due to sandstones having a larger $\varPhi (0)$ value, see Table 1). Meanwhile, the value of $\varTheta (0,t)$ required to attain $K_{r}(0,t)\approx 0.1$ at the top boundary is surprisingly high in loams, a little smaller in sandstone and smaller still in foams. Correspondingly the time at which $K_{r}(0,t)\approx 0.1$ is much larger than the time at which $\varTheta (0,t)\approx 0.1$ in soils. This is necessarily the case, because as already mentioned, $K_{r}\approx m^2 \varTheta ^{1/2+2/m}$ here. Hence, at times when $\varTheta$ is still comparatively small (e.g. $\varTheta \approx 0.1$), and remembering $m<1$, it must be the case that $K_{r}$ is much smaller than $\varTheta$. Thus, for $K_{r}$ to reach a value $K_{r}\approx 0.1$ requires substantially more time. On the other hand, for loams, the time at which $K_{r}(0,t)\approx 0.1$ is of similar order magnitude to the time (at least as predicted via the purely diffusive model) at which $\varTheta (0,t)\approx 1$, but somewhat smaller than that for sandstones. Again this follows from the formula $K_{r}\approx m^2 \varTheta ^{1/2+2/m}$: given loams tend to have a small *m* values, a value $K_{r}\approx 0.1$ can be achieved without requiring a particularly small value of $\varTheta$. Meanwhile, for foams, the time at which $K_{r}(0,t)\approx 0.1$ is well over an order of magnitude smaller than the time at which $\varTheta (0,t)$ becomes close to unity.

**Table 2:** Table showing time for moisture accumulation

Porous medium	$t_{\varTheta (0,t)\sim 0.1}$	$\varTheta _{K_{r}(0,t)\sim 0.1}$	$t_{K_{r}(0,t)\sim 0.1}$	$t_{\varTheta (0,t)\sim 1}$
Node-dominated foam	0.0079	0.2154	0.0365	0.7854
Channel-dominated foam	0.0018	0.3162	0.0328	0.5829
Silt Loam	$2.5292~\times 10^{-6}$	0.8009	0.0262	0.0702
Guelph Loam	$6.1800~\times 10^{-6}$	0.6799	0.0150	0.0723
Hygiene Sandstone	$7.8176~ \times 10^{-6}$	0.4611	0.0019	0.0316

### Estimating $x_{\max }$

We know that for the soils using [9] or using channel-dominated foam drainage [20], the early-time solution terminates at some point $\eta _{\max }$, beyond which $\varPhi = 0$. We can estimate at which depth $x_{\max }$ this occurs (or equivalently given the depth to which moisture penetrates, we can estimate the time for which infiltration or imbibition has been ongoing).

We know $x_{\max }=\eta _{\max }t^{(N+1)/(N+2)}$ which in the case of soils now becomes $x_{\max }=\eta _{\max }t^{(2+3m)/(2+5m)}$. Based on the power law, for $t\ll 1$, loams (smaller *m*) show slower growth in $x_{\max }$ than sandstones (larger *m*), which are then slower in turn for channel-dominated foam drainage ($x_{\max }=\eta _{\max }t^{3/5}$). These effects are offset by loams having a larger $\eta _{\max }$ than sandstones do (see Table 1).

Note that the node-dominated foam drainage case is a little different from the others in that there is no finite $\eta _{\max }$ and hence no finite $x_{\max }$ at which $\varTheta$ is exactly zero. Figure 2 however makes it clear that $\varPhi$ is negligibly small for any $\eta$ greater than about 4, hence any *x* greater than about $4 t^{1/2}$.

In the case of a soil, the distance that moisture penetrates satisfies $x_{\max }=\eta _{\max }t^{(2+3m)/(2+5m)}$, as we have said. If in a given soil sample we were to measure $x_{\max }$ versus time *t*, we could use these measurements to estimate the soil specific parameter *m*. Relative diffusivity RD ($D_{r}\approx m^{-1}(1-m)\varTheta ^{1/2+1/m}$), relative hydraulic conductivity RHC ($K_{r}\approx m^{2} \varTheta ^{1/2+2/m}$) and the soil water retention curve SWRC ($H_{+}\approx \varTheta ^{-(-1+1/m)}$) expressed here in suitable dimensionless form, then follow. The SWRC rearranges to $\varTheta \approx H_{+}^{-m/(1-m)}$. The capillary suction head $H_{+}$ can be related to a pore size *r*, these two quantities being inversely proportional, i.e. $H_{+}\propto r^{-1}$. It then follows that $\varTheta \propto r^{m/(1-m)}$. We can then relate this to a distribution of pore sizes *f*(*r*), on the grounds that (by definition) $\varTheta = \int f(r)\,\mathrm {d}r$. It follows that $f(r) \propto r^{(2 m - 1)/(1-m)}$. Here we are specifically considering the small $\varTheta$ limit and hence the small *r* limit: pores that are much smaller than average have the strongest capillary suction, so they are the first to fill with any moisture. Note that sandstones (which have *m* close to unity) have extremely small *f*(*r*) in the small *r* limit. Loams on the other hand (with *m* close to 1/2) tend to have not quite such a small *f*(*r*) in this small *r* limit. In summary, by measuring $x_{\max }$ versus *t* and inferring the soil-specific parameter *m*, we can infer information about pore size distribution. The analysis here only however considers pores much smaller than average. The average pore size in a loam might of course be very different from the average pore size in a sandstone, but that effect is scaled out in the dimensionless formulation employed here. Moreover (as alluded to in Sect. 2), the parameter *m* also furnishes information about how pore size distribution behaves for pores that are much larger than average. However, such pores are only ever filled as full saturation is approached, so details of how their sizes are distributed does not impact the early-time, low saturation limit considered here.

## Conclusion

The early-time diffusion equations obtained for moisture propagation in foams and soils may be either linear or nonlinear equations based on whether we have constant or variable diffusivity functions describing the dominant capillary-driven transport of moisture. Either way, solutions are available in similarity form. Moreover, by casting the equations in dimensionless form we have been able to compare and contrast foams and soils. We have given the analytical solution for the node-dominated foam drainage, and the numerical solution for drainage in channel-dominated foams and imbibition in soils, the latter based on the diffusivity function of [9]. The numerical similarity solutions were obtained via the shooting method.

The foam drainage similarity solutions have been compared with those obtained for soils. Even though these are early-time solutions, in all cases it was found that the profiles are similar in some regards to the long-time solutions, in particular the soils approaching the “dry region” (the limit as $\varTheta \rightarrow 0$) abruptly, just as was obtained for long-time travelling wave solutions. In fact, the nonlinear cases (channel-dominated foam drainage and drainage in soils) fall to zero moisture content at finite distances $x_{\max }(t)$, but it is only the soils that exhibit abrupt singularities in the profile shape as $\varTheta \rightarrow 0$. This behaviour follows from the diffusivity function decaying to zero rapidly with falling $\varTheta$ in the case of soils.

We examined how the evolving moisture content at the boundary of a system $\varTheta (0,t)$ develops as we deliver a known imbibition flux. In foams, we found that this boundary moisture content value is greater in channel-dominated foams (growing like $t^{2/5}$ for $t\ll 1$) than in node-dominated foams (growing like $t^{1/2}$). In soils, the top boundary moisture content depends on a soil-specific parameter *m* which is close to unity for sandstones, but smaller than that for loams. The moisture content required at the boundary to deliver a known flux grows like $t^{2m/(2 + 5m)}$ for small *t*. This quantity increases as the value of *m* decreases, indicating that $\varTheta (0,t)$ is faster growing for loams than for sandstones. Overall though, the $\varTheta (0,t)$ value tends to be greater in soils than in foams.

Systems that have faster growing $\varTheta (0,t)$ at the top tend to terminate at lower $x_{\max }$ values (i.e. at lesser depth). For soils $x_{\max }$ scales like $t^{(2 + 3m)/(2 + 5m)}$ for $t\ll 1$. This corresponds to comparatively slow growth in $x_{\max }$ for loams (smaller *m*) but faster growth for sandstones (*m* close to unity), albeit still not as fast as channel- or node-dominated foams, for which the respective scalings are $t^{3/5}$ and $t^{1/2}$.

We emphasise that the various solutions described here are only early-time ones and we have provided estimates of dimensionless times at which significant hydraulic conduction flux (i.e. significant effect of gravity) will start to develop, indicating that infiltration is no longer a pure capillary-driven imbibition process. Significant hydraulic conduction tends to happen sooner in soils than in foams, and sooner still in sandstones than in loams. Despite the faster early-time growth in $\varTheta (0,t)$ for loams compared to sandstones, in the case of sandstones, the hydraulic conduction contribution to the flux tends to grow faster. This is because hydraulic conduction flux is a more rapidly increasing function of moisture content in sandstones than in loams, meaning that significant hydraulic conduction flux can be realised for sandstones at a more modest moisture content. Of course at even longer times, the early-time solutions obtained here break down altogether and we recover the long-time travelling wave solutions already considered by [1], which for the unit infiltration flux considered here, turn out to give $\varTheta (0,t) \approx 1$ at long times. How the system transitions between the early-time and late-time behaviours is a question which in general would need to be addressed numerically, by a methodology like that already developed by [4].
